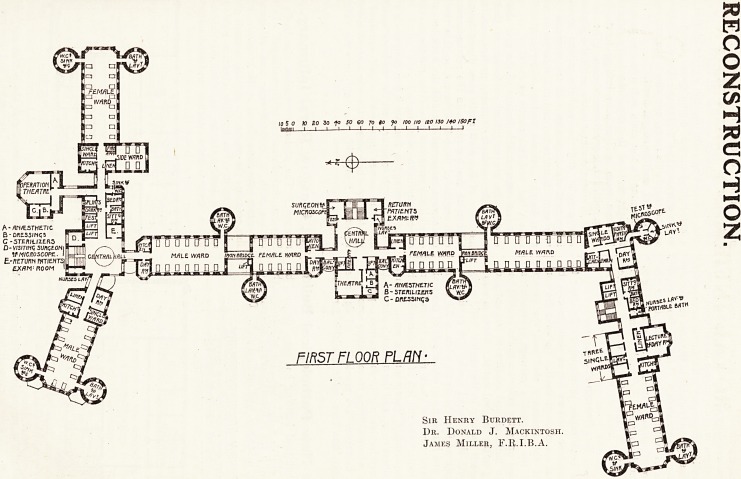# Some Special Features of Importance

**Published:** 1914-11-28

**Authors:** 


### THE ROYAL INFIRMARY, GLASGOW.*

### II. Some Special Features of Importance.

To understand the plan upon which the Royal Infirmary, Glasgow, has been constructed it is essential to keep clearly in mind, (1) that the general ground plan of the main ward blocks was arbitrarily fixed by the managers, and left neither the technical experts nor the architect any freedom as to the position the buildings should occupy upon the present site; (2) Scottish hospitals are administered upon a system not to be found at present in any English hospital. It provides that every unit of construction—*i.e.*, (*a*) the medical wards, (*b*) the surgical wards, and (*c*) the special departments—shall be self-contained both as to executive control and medical service. That is to say, as the plans show, taking the first floor as an example, there are on the surgical side three large wards, two containing twenty-nine beds (of which one is in a single ward), and one for women containing sixteen beds, with a single-bedded ward and a four-bedded ward also, making together twenty-one female beds, or a total of fifty male and female beds in each floor unit.

### The Surgical Ward Pavilions.

Each important ward has a kitchen and also a day room, the latter being a most attractive, airy apartment, which constitutes a useful and popular feature of this hospital. A sink and w.c. have been provided for the small wards, as shown on the plan. Each floor has ample lavatory and bath accommodation, a clinical room, a room for patients who return for examination after discharge, as is the practice in Scottish hospitals, a room for splints, a dark room, a test room, with ample accommodation for the linen, and other housemaids' requirements. In addition to this the unit includes a flat, which provides most comfortable apartments for the resident medical officer in charge of the complete surgical unit on each floor.

These fifty surgical beds on each floor are arranged in three pavilions, each of which is cut off from all the other buildings, and each provides the maximum facilities in regard to cross-ventilation, light, and air. A separate operation unit is provided for every fifty beds on each of the five floors. With a view to secure the maximum of hygienic advantage the operation theatres have been isolated from the other buildings, and are cut off by a communicating corridor. Attached to the operation theatre are an anæsthetic room and rooms for dressings and sterilisers. Provision has also been made in a recess for the fire apparatus.

The arrangements just described are repeated on each of the five floors of the surgical block above the ground floor. An arrangement has been made whereby, in case of fire or other emergencies, beds can be moved from one block to another across fireproof passages, so that there is a possible means of escape on each floor from one department to another. A feature of this and the other pavilions is the central hall, where the main staircase is placed, with a nurses' lavatory in one corner, and to the right of the staircase the visiting surgeons' and microscope room.

### The Medical Ward Block.

This block is approached by the main doorway, on passing which we find ourselves in a spacious entrance hall. To the right are folding doors leading to the electrical department. Passing along the left corridor we have on the right cloak-rooms for female and male students, with lavatories. Next a fine suite of rooms with a waiting room, originally intended for the Dorcas department, which has now been transferred to the basement, which, it may here be mentioned, is not a basement at all in the old sense, but simply a lower ground floor which the nature of the site has favoured, where excellent accommodation, equal to most if not all of the accommodation to be found on the ground floor, has been provided. Returning to the entrance hall, we have on the left corridor, beyond the cloakrooms just mentioned and immediately to the left, the main staircase, then the lifts, then a linen store and workrooms, where air and light are essential requisites, with housemaids' and other domestic accommodation.

### The Medical Ward Units.

The arrangement of the wards proper and all that appertains to their administration on each of the five upper floors of the medical block are identical. To the right of the corridor from the staircase and lifts is a large female ward of sixteen beds, with a bath and lavatory in one tower, w.c.s and slop sinks in the other. A ward kitchen, with three small wards, a bathroom and w.c., a linen store, a day and lecture room, complete the accommodation here.

Turning from this ward through the cut-away we have the staircase and lifts on the left, and on the right a nurses' lavatory and portable bath, and the flat containing a sitting room, bedroom and bathroom *en suite* for the medical resident. Next there is the visiting physicians' and microscope room, a test room, occupying one-half of the tower, the rest being devoted to w.c., sink, and lavatory accommodation for the two single male wards. Then there are the day room, ward, kitchen, and linen store, with a large male ward of eighteen beds, bath, lavatory, and w.c. accommodation, as shown on the plan.

The medical ward unit on each floor contains twenty male beds and twenty-one female beds.

### The Central Block for Special Diseases.

The accommodation for the special departments is arranged on separate floors. The ward unit on

* Article I. appeared on November 14, p. 155.

each floor is completely cut off by a cross-ventilating passage, and contains ten beds in each of two large wards. Taking the special departments in order: On the first floor there are the gynæcological wards, containing twenty beds. Here there are no small wards, and each of the large ones has its own day room and kitchen with separate bath and lavatory accommodation in the tower. They are separated by a central hall of communication, where are to be found the surgeons' and microscope, test and returned patients' examination rooms, nurses' lavatory, and lifts. The operation theatre is placed in the front block with a good north light; an obscured glass partition with a fanlight above forms a vestibule, cutting off the amphitheatre from the central hall. An anæsthetic room, a sterilising room, and a room for the storage of surgical dressings are placed conveniently to the theatre and isolated from the central hall. These rooms are well lighted. On the second floor are the Burn wards. They comprise a ward for males of ten beds, a ward for females of ten beds, and a single ward containing a permanent bath-bed, making twenty-one beds in all. They have the same adjuncts as the gynæcological wards, but no theatre.

On the third floor are the throat and ear wards, which consist of a ward of ten beds and a single ward with one bed for males, and a ward of ten beds and a single ward for females, making twenty-two in all, with the necessary bath, lavatory, and w.c. accommodation. There are also a ward kitchen, test and linen rooms, nurses' lavatory, dark-room, the visiting surgeons' and microscope room, and a dark-room for otological and laryngeal examinations with lamps. The throat and ear wards have an excellent operation theatre with the arrangements already described.

On the fourth floor are the skin wards. They consist of a male ward containing ten beds and a single-bedded ward, a female ward with ten beds and an isolation ward with one bed, available for use for male or female patients at discretion. A day room has been provided adjacent to each of the large wards, with linen and test rooms as on the floor below, together with the visiting surgeons' and microscope room.

The resident medical officers are accommodated on each alternate flat, as this arrangement has permitted the theatres to be better lighted and of greater height. The four blocks of wards for special diseases have a resident in charge of each two of these units, one resident taking the gynæcological and burn wards, and the second resident the throat and ear and skin wards respectively.

Ample accommodation exists for the treatment, when desired, of a large number of the patients in the open air on the roof and in the interspaces which separate the medical and surgical ward units from the special ward units on either side of the hospital respectively.

It will be manifest to hospital administrators and managers familiar with the plan of a modern hospital how good are the arrangements whereby on each floor a separate surgical unit, a separate special diseases unit, and a separate medical unit are placed, all of which—though the several pavilions of each unit are cut off from all the other buildings and each provides the maximum facilities in regard to cross-ventilation, light, and air—can be readily worked with a minimum strain upon the nursing and medical staff, whilst the whole hospital can be inspected floor by floor by the administrative officers with the maximum of facility and despatch.

### The Call for Administration.

It was a very real pleasure and privilege to inspect the whole of this fine hospital and to study it fully from every aspect—hygienic, structural, medical, and administrative. The arrangement on the Scotch system already described enables the management to control this hospital, so far as its wards and the medical, surgical, and clinical aspects of its work are concerned, with the maximum of ease and effectiveness. The builders and architects, designers and experts have done their work admirably, but a fine hospital with 700 beds (which Germany, out of a large experience, has come to recognise is about the maximum number a great hospital should contain if it is to be smartly administered) must have for the whole of it a male and female director or directors of the highest intelligence, the widest knowledge and experience, the maximum of health and driving force, and the most splendid gifts and spirit of devotion to the work over which they will be called upon to preside. Taking the world as a whole, and considering carefully every opportunity, every choice of a field of labour or occupation for men and women of the highest intelligence and the noblest character, we venture to declare, with nearly fifty years' experience behind us, that the most satisfying, inspiriting, uplifting, and Christ-like work open to human beings as members of the higher civilisation is undoubtedly continuous service in a great hospital of the first class. Anyone with knowledge who inspects thoroughly from end to end the Glasgow Royal Infirmary, must realise that, if it is to fulfil its purpose continuously and adequately, it is essential that the old plan of administering the affairs of this institution, and many of the methods which prevailed in other days, under other circumstances, must be entirely eliminated. To succeed to the maximum extent in the great work which this hospital has been erected to undertake, it is essential that from the highest officer to the humblest worker, in each and all of the departments which collectively constitute the Royal Infirmary, Glasgow, to-day, there must be the right spirit, the necessary knowledge, absolute devotion, and continuous effort. Where these qualities are present, they never fail to secure that the whole establishment, from top to bottom, throughout every department, shall present the maximum of smart efficiency and the best results which the trained and experienced worker can produce day by day.

This is an occasion when it may be useful to call attention to the fact that a fine hospital of the modern type requires, and to succeed must obtain, a system of administration which never sleeps and never passes inefficient work or slackness, or permits untidiness, much less slovenliness, to find any place whatever in any single portion of the institution from one end to the other. We have great confidence in the ultimate successful administration of this fine hospital. This confidence is based upon a close observation and study of the nursing staff, upon our observation of the gentlemen who have the high honour and privilege to occupy the resident posts at this hospital at the present time, and upon the fact that many of the honorary staff are younger men with keen scientific interests, a commendable pride in the position which they occupy, and a due sense of their individual responsibilities in the special circumstances with which the plan and opening of the new hospital endows them. But the managers, in conjunction with the present medical superintendent and matron, have a very serious difficulty to face in wisely determining what exactly is to be the system of administrative control, by and through which alone the maintenance of absolute efficiency can be everywhere introduced and continuously enforced in the daily routine and working of a vast establishment like theirs. Some people hold that it is desirable to have more than one high official in the position of director. Our experience convinces us that the real secret of success in this direction is to be obtained by the arrangement of each department under responsible assistants of the best type, trained for the work, and then to procure as director a gentleman who has given evidence, not only of his knowledge, but of his power to deal with a vast establishment like that of the Glasgow Royal Infirmary in such a way that, the atmosphere of the whole place is unmistakably pleasant, efficient, and free not only from sleepy indifference, but from all personalities and disagreements. These can never find a place where the chief directors, both men and women, love their work and take such a pride and pleasure in it and its efficiency in results that, it is regarded by them as a privilege and delight to continue in office and to work continuously to make their institution, admittedly, as perfect, as instructive, and as useful as any great hospital throughout the world.

Consider the modern hospital from the economic, scientific, medical, surgical, nursing, educative, whatever point of view you will, and you will become aware that it is not possible to compare fairly and adequately the work of one hospital with another, unless the factors of comparison are resolved into sections, and the particulars and facts regarding each section are made exhaustive and complete. Then the facts of each section can be properly compared with the section of any other equally large and important establishment, and when the whole result is totalled up it is possible, in endeavouring to come to an appreciation of relative merit, to make allowances for differences which do and always must exist between one great hospital and another. It is natural that we should have these considerations impressed upon us after many hours on separate days spent in the inspection of a fine hospital, like the one we are here dealing with, because of our sympathy for the workers, who have the responsibility for its administration, begotten of many years' work of a similar character in circumstances which were not facilitated, as they are nowadays, by modern equipment and the splendid efficiency which characterises many of the fittings, appliances, and structural details which have eventuated out of a continuous effort to advance in the treatment and care of the sick. Such zeal has characterised hospital effort in this country and many other countries during the last forty years of continuous progress.

### The Executive and other Departments.

These departments include the Nurses' Home, the laundry, kitchen, dispensary and drugs department, and the stores. The kitchen has been placed on the sixth floor. It is well lit and well ventilated. It contains the scullery, kitchen, maids' dining room, and abundant accommodation for (1) milk and butter, (2) cook's stores, (3) pastry, (4) cold meat, (5) vegetables, (6) fish, and (7) other necessary stores for the kitchen. There is also an excellent pantry. In connection with it there is a bed and sitting room for the cook and five double bedrooms for the kitchen staff, together with bathroom and lavatory accommodation. A linen room, housemaids' closet and lavatory, and a room for trolleys are also shown on the plan. Convenient and adequate service lifts have been provided to the various departments.

On the lower ground floor provision has been made in the simplest and readiest manner for the efficient administration of the various departments, which will facilitate the easy working of the entire hospital. On entering the gate from Vicar's Alley a weighing-plate is placed on the left, in order that all carts with coals, etc., may be weighed and checked before the delivery of the goods. In the basement of the front block (facing Cathedral Square) the blanket store, uniform store, linen store, and lavatory for linen-room staff were placed, but these seem to have been transferred to similar accommodation on the ground floor proper. Taking the basement from south to north the visitor will find a large milk store, refrigerator room, and stores for (1) butcher's meat, (2) bread, milk, flour, etc., (3) general groceries, (4) crockery and ironmongery, (5) steward's office and his waiting room. The latter are placed adjacent to the lift, which runs direct from the basement to the kitchen on the top of the building. Next to the lift are a service room and the porters' dining room, so that their food is brought straight from the kitchen with the greatest economy of service and the maximum of comfort to the staff. Then come the porters' sitting room, and cloak, boot, and trolley rooms.

Coming to the lower ground-floor entrance hall, we find the staircase occupying the centre, with the coal house and ice store on either side of it. On the left is the patients' lift, leading to the wards, with the entrance to the subway leading from the admission block at the gate house. Passing through

### ROYAL INFIRMARY, GLASGOW:

GROUND PLAN.

Sir Henry Burdett.

Dr. Donald J. Mackintosh.

James Miller, F.R.I.B.A.

### RECONSTRUCTION.

FIRST FLOOR PLAN.

Sir Henry Burdett.

Dr. Donald J. Mackintosh.

James Miller, F.R.I.B.A.

the hall we find the wine cellar and empty-bottle store, and further on a large dining room for cleaners and wardmaids, with its service room adjacent and connected by a lift with the kitchen.

Finally, there is a dispensing department, comprising a well-lit dispensary with ample storage for surgical dressings, and outside in the area temporary provision for inflammables. There is also a good-sized room in which is kept some plant for the manufacture of aërated waters. It seemed to us to need thorough overhauling and reorganisation. There is also a store for glassware and for stationery.

### The Ground Floor.

Care has been taken in planning this floor to arrange that each unit as far as possible may be separate and complete in itself. To meet the convenience of the honorary staff and the general working of the hospital a carriage approach has been provided up to the entrance to the central hall of the main building, and another to the entrance hall leading to the medical wards. There is also a door to the left leading to the central hall, which connects with the surgical block. On passing the main entrance we find the porter's box in the centre of the hall, and behind it, on either side of the main staircase, lavatories and cloak rooms for male and female students. On the left of the entrance is the matron's department, comprising a small waiting room, a business room, and the matron's office, with a private corridor leading to the matron's private apartments, where a dining room, sitting room, bedroom, and bathroom are provided. This latter accommodation has been made available for other purposes owing to changes introduced by the managers. They consist of the provision of two flats on the sixth and seventh floors, one for the matron and one for the medical superintendent. Passing on we find the kitchen service lift directly connected with the service room for the supply of food, and a well-lighted nurses' dining room placed opposite to the entrance to the Nurses' Home. Further on are the chaplain's room, opposite to the entrance to the chapel, and the chapel itself.

Returning to the main entrance and passing to the right we find the superintendent's office and business room, and a lobby leading to the board room, with lavatory accommodation. Then comes a lift in direct communication with the kitchen, a service room, and the residents' dining room. A lobby next communicates with the residents' lavatory and billiard room, and in the corner is placed a flat for the resident medical officer, containing a sitting, bed, and bath rooms *en suite*.

It is a commendable feature of the plan that the service lifts are so placed as to secure that the food of the residents, nurses, porters, and other members of the staff shall pass direct from the kitchen to the hot plates in the service rooms of each section of the establishment, so that the food can always be served hot and the work be carried on with the minimum of service and the maximum of economy in the working.

In planning this hospital it has been made a cardinal principle to secure that the residents' quarters, flats, and nurses' quarters are self-contained and isolated, by which means the administrative departments are completely shut off from the patients' wings.

## Figures and Tables

**Figure f1:**
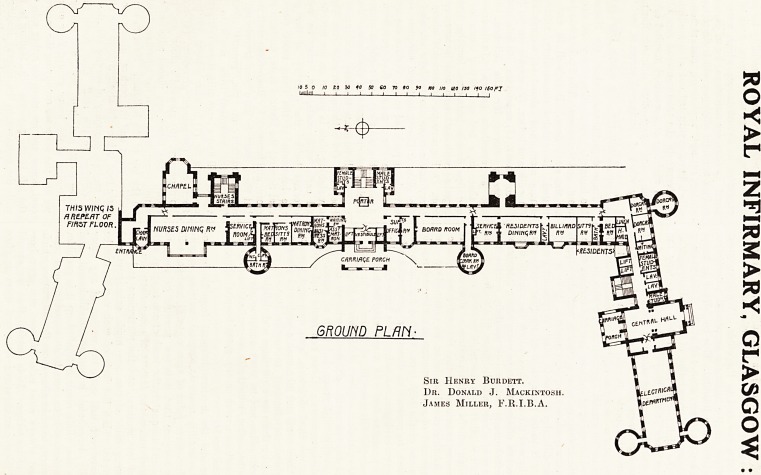


**Figure f2:**